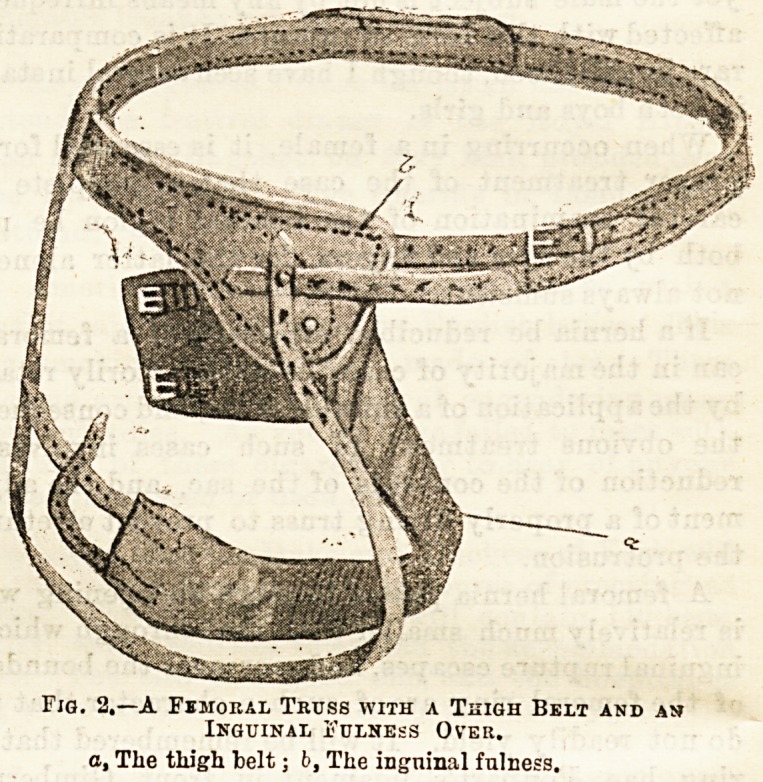# On the Treatment of Femoral Hernia

**Published:** 1896-03-21

**Authors:** W. McAdam Eccles

**Affiliations:** Assistant Surgeon to the City of London Truss Society and to the West London Hospital; Surgeon to the St. Marylebone General Dispensary, &c.


### Medical Progress and Hospital Clinics.

[*The Editor will be glad to receive offers of co-operation and contributions from members of the profession. All letters should be addressed to* The Editor, at the Office, 428, Strand, London, W.C.]

### ON THE TREATMENT OF FEMORAL HERNIA.

By W. McAdam Eccles, M.S.Lond., F.R.C.S.Eng, Assistant Surgeon to the City of London Truss Society and to the West London Hospital; Surgeon to the St. Marylebone General Dispensary, &c.

### I.—Reducible Femoral Hernia.

Although femoral hernia is more common in women, yet the male subject is not by any means infrequently affected with this form of rupture. It is comparatively rare in childhood, though I have seen several instances in both boys and girls.

When occurring in a female, it is essential for the proper treatment of the case that a complete and careful examination of the femoral region be made both by the eyes and fingers, for the latter alone are not always sufficient.

If a hernia be reducible, especially if a femoral, it can in the majority of cases be satisfactorily retained by the application of a suitable truss, and consequently the obvious treatment of such cases involves the reduction of the contents of the sac, and the adjustment of a properly-fitting truss to prevent a return of the protrusion.

A femoral hernia passes through an opening which is relatively much smaller than that through which an inguinal rupture escapes, and moreover the boundaries of the femoral ring are of such a character that they do not readily yield. It will be remembered that this ring has Poupart's ligament in front, Gimbernat's ligament with its sharp edge on the inner side, the horizontal ramus of the os pubis covered by the pectineus muscle behind, and on the outer side are the femoral vessels, which can be but little displaced. These conditions not only predispose to some gripping of the protruded parts, but further to their strangulation, and moreover render reduction even in a reducible hernia sometimes difficult to accomplish. There are some points of practical importance to be observed in employing taxis in these cases. Firstly, I would venture to emphasise the fact that a very considerable degree of force—using that term in a surgical sense—may be applied in dealing with hernia provided that it is directed in the proper way, and with a definite and legitimate end in view. Healthy bowel and omentum, as we may consider that which has only been quite recently protruded to be, can stand severe pressure without any harm arising; but if strangulation happen to have been present for some hours only, a much less degree of force is justifiable, because it is in such cases that the gut is likely to be bruised or even lacerated. Secondly, pressure must be applied and maintained in a firm, steady, and continuous manner. Intermittent kneading is to be deprecated, for it is worse than useless, since it gives the patient unnecessary pain, is apt to bruise the parts, and but seldom secures the return of the contents of the sac. It is a fact well worthy of notice, and which, no doubt, most have observed, that cases where little or no discomfort is experienced by the patient during taxis are usually those in which reduction will not follow, for it seems that the movement of the protruded parts towards the abdominal cavity gives rise to a considerable degree of pain in the larger number of instances. Thirdly, care must be taken that the swelling itself is fully and fairly grasped by the fingers of the surgeon. Not infrequently patients complain much of the pinching of the skin which they experience while efforts are being made to reduce the hernia. If, however, the tumour be surrounded at its base, but little of this unpleasant sensation will be felt by the patient. Lastly, the direction of the pressure is very important. A femoral hernia, when discovered, has usually already passed down the crural canal and through the saphenous opening, and may, indeed, be somewhat compressed by the edges of the latter. The protrusion will thus have to be primarily reduced through this opening by pressure, which will need to be directed backwards. This being accomplished, the contents of the sac have to pass upwards through the canal and the ring into the abdomen; in this stage, then, the direction of the force must be altered to that of almost directly upwards, the fingers being placed below the swelling, and pushing it in this direction.

The patient during taxis should, of course, be lying supine, but I do not consider the position of the lower limb on the affected side has much influence upon the ease with which reduction is effected. The unyielding nature of the parts does not allow of much change, whether the thigh be flexed or extended, rotated inwards or outwards. Personally I prefer extension with some external rotation. The surgeon should stand on the same side as the hernia, and facing the patient's feet. It is advisable to use both hands, the better to satisfactorily grasp the rupture. A hernia which defies reduction on one day may often be quite easily reduced on another, and more especially if taxis be employed after a period of rest in the recumbent position. This is only in simple irreducible hernia.

Having satisfactorily returned all the contents within the abdomen, the next step is to apply a suitable and efficient truss. Variety in the size and shape and other details of a femoral truss may be seen in almost any truss-maker's catalogue. Some of these are more or less good, others are decidedly bad. The anatomy of the parts must, one would think, have either been unknown or disregarded by the inventors of these latter. Of all trusses a femoral requires to fit with accuracy and precision.

The accompanying drawing (Fig. 1) is a very faithful representation of the form of femoral truss which I think is most generally of use. It is usual to make the spring of a femoral truss lighter than that used for an inguinal, and the pad again is smaller than in the latter, and faces more directly upwards, since the femoral ring in the erect posture is almost horizontal. The first point in adjusting a truss is to take a measurement in order to determine the size required. This would seem to be a simple matter, but it is perhaps the one detail over which a good deal of difficulty and uncertainty arises. This is probably owing to no very clear or precise method as to how the measure should be obtained being described. A tape measure should be drawn directly around the pelvis on a level which is midway between the highest part of the crest of the ilium and the top of the great trochanter, the limbs being neither abducted nor adducted. No regard need be paid to the position of the hernial aperture, for in my experience this leads to inaccurate measurements in many cases. For a single truss the above measurement is, if correct, sufficient; but for a double truss, owing to its having no part which will

FIG. 1.—AN ORDINARY FEMORAL TRUSS.
a, The shoulder of the truss; b, The understrap; c, The point where the steel spring should terminate.

stretch at all, it is requisite to add one inch to the actual measurement in order that the instrument may fit comfortably. If a femoral truss be too large—which is the common fault—it is certain that the pad will press on the spine of the os pubis, and thus cause the greatest discomfort. On the other hand, if the truss be too short the pressure will be so great that the patient will not bear it and will discard the truss.

In ordering a truss from an instrument maker, besides giving the measurement indicated, it is necessary to say whether it is for an inguinal or a femoral rupture, whether it is to be a single truss, and if so, for which side, or a double instrument, and it is well, in addition, to state whether the patient is of the male or female sex, and whether the spring of the truss is to be of the ordinary strength, or weaker or stronger than usual. It must be remembered that the understrap is an essential part of the truss. This strap should be attached to the spring just behind, or even at the shoulder, and not considerably farther back, as it is so often placed. Moreover, I prefer to fasten it to the upper button on the pad, for it will then tend to give this an inclination to face upwards against the ring, which is of advantage. Such a truss as is here spoken of will in the larger number of cases satisfactorily retain a femoral rupture. It is, however, the experience of many to have seen patients whose herniæ

FIG. 2.—A FEMORAL TRUSS WITH A THIGH BELT AND AN INGUINAL FULNESS OVER.

a, The thigh belt; b, The inguinal fulness.

are so large that an ordinary femoral pad fails to keep them reduced.

It must be remembered that these protrusions have a tendency to travel upward on to the abdomen in front of Poupart's ligament, and no doubt in some cases they escape from beneath the pad, owing to this being displaced above the area of the femoral ring. To obviate this, it is best to prolong the pad downwards, and to employ a belt of flexible material to pass around the thigh and to buckle on the outer side, the femoral pad at the same time being surmounted by the addition of an inguinal fulness. This variety of truss is depicted in Fig. 2. The question of the radical cure of a femoral hernia will be discussed at another time when the treatment of irreducible femoral herniæ is dealt with.

## Figures and Tables

**Fig. 1. f1:**
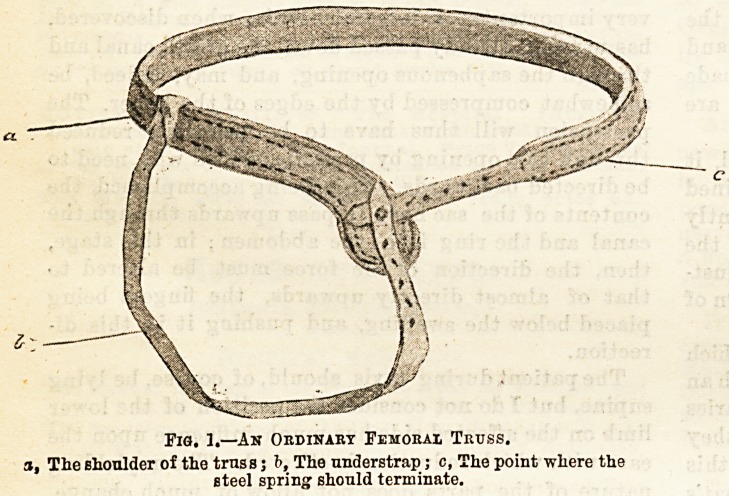


**Fig. 2. f2:**